# The PanAM study: a multi-center, double-blinded, randomized, non-inferiority study of paracetamol versus non-steroidal anti-inflammatory drugs in treating acute musculoskeletal trauma

**DOI:** 10.1186/1471-227X-13-19

**Published:** 2013-11-20

**Authors:** Milan L Ridderikhof, Philipp Lirk, Niels W Schep, Anneke Hoeberichts, Wilhelmina T Goddijn, Jan SK Luitse, E Marleen Kemper, Marcel G W Dijkgraaf, Markus W Hollmann, J Carel Goslings

**Affiliations:** 1 Department of Emergency Medicine; Academic Medical Center, PO Box 22660, 1100 DD, Amsterdam, The Netherlands; 2 Department of Anaesthesiology; Academic Medical Center, PO Box 22660, 1100 DD, Amsterdam, The Netherlands; 3 Trauma Unit Department of Surgery; Academic Medical Center, PO Box 22660, 1100 DD, Amsterdam, The Netherlands; 4 General Practice “Health Care Center Gein”, Wisseloordplein 50, 1106 MH, Amsterdam, The Netherlands; 5 Department of Pharmacy; Academic Medical Center, PO Box 22660, 1100 DD, Amsterdam, The Netherlands; 6 Clinical Research Unit; Academic Medical Center, PO Box 22660,1100 DD, Amsterdam, The Netherlands

**Keywords:** Wounds and injuries, Strains and sprains, Contusions, Analgesia, Pain, Paracetamol/acetaminophen, Anti-inflammatory agents, Non-steroidal, Costs and cost analysis

## Abstract

**Background:**

Acute musculoskeletal trauma, including strains, sprains or contusions, occur frequently. Pain management is a crucial component of treatment. However, there is no convincing evidence which drug is superior in managing pain in these patients. The aim of the PanAM Study is to compare analgesic efficacy of three strategies of pain management: paracetamol, diclofenac, or a combination of both in patients with acute musculoskeletal trauma.

**Methods/design:**

The PanAM Study is a multi-center, double blind randomized controlled trial with non-inferiority design. Included are adult patients presenting to an academic, urban Emergency Department or to a General Practice with acute, blunt, traumatic limb injury. In total, 547 patients will be included using a predefined list of exclusion criteria, to be allocated by randomization to treatment with paracetamol + placebo diclofenac, diclofenac + placebo paracetamol or paracetamol + diclofenac. The hypothesis is that paracetamol will not be inferior to treatment with diclofenac, or the combination of both. Primary outcome will be between-group differences in decrease in pain, measured with Numerical Rating Scales at baseline and at 90 minutes after study drug administration. Secondary outcomes are Numerical Rating Scales at 30 and 60 minutes and measured frequently during three consecutive days after discharge; occurrence of adverse effects; patient satisfaction and an analysis of quality of life and cost-effectiveness. Recruitment started July 2013 and is expected to last a year.

**Discussion:**

With this multi-center randomized clinical trial we will investigate whether treatment with paracetamol alone is not inferior to diclofenac alone or a combination of both drugs in adult patients with acute musculoskeletal trauma. The main relevance of the trial is to demonstrate the benefits and risks of three commonly used treatment regimens for musculoskeletal trauma. Data that lead to the prevention of severe Non-Steroidal Anti-Inflammatory Drugs-related adverse effects might be gathered.

**Trial registration:**

Dutch Trial Register (http://www.trialregister.nl): NTR3982.

EudraCT database (http://www.clinicaltrialsregister.eu): 201300038111.

## Background

Musculoskeletal trauma has a high incidence. In The Netherlands, in 2011, 3.3 million injuries were treated medically in a total population of 16.7 million [1]. Taken per capita, this density is approximately the same in the United States. In 2006/2007, more than 61.2 million patients with musculoskeletal injuries were treated annually in the United States in a total population of 300 million [2]. Most injuries concern soft tissue, such as strains, sprains and contusions, also referred to as acute musculoskeletal syndromes [3]. Strains and sprains is a collective term for muscle and ligament injuries without dislocation or fracture. A contusion is a haemorrhage (usually in the skin) resulting from a direct trauma. These injuries are often treated in the Emergency Department or in General Practice. Pain management in the acute phase is a crucial part of treatment and can be non-pharmacological (RICE: Rest, Ice, Compression, Elevation) and/or pharmacological [4,5]. The treating physician prescribes paracetamol (acetaminophen) or Non-Steroidal Anti-Inflammatory Drugs (NSAID’s), or patients use these drugs without prescription, as they are available over the counter. It is unclear, however, whether NSAID’s have any additional value for the treatment of pain in patients with an acute musculoskeletal trauma [3]. Because of supposed efficacy, costs and side effect profile, several guidelines mention that paracetamol might be as appropriate as NSAID’s, however the supporting evidence is scarce [6,7]. Therefore, there is a need for supportive data for the effectiveness of paracetamol compared with NSAID’s in managing pain after acute musculoskeletal trauma.

Besides the question whether paracetamol is not inferior to NSAID’s in patients with acute musculoskeletal trauma, it is important to realize that the use of both drugs can have detrimental side effects. Paracetamol is an analgesic and antipyretic, while the exact mechanism of action is not known. It should be used with attention to dosage, as paracetamol overdose is the leading cause of acute liver failure in the United States and other Western countries [8]. It is widespread available and frequently, paracetamol is part of combination preparations. Because many patients have difficulties identifying prescription and over the counter products containing paracetamol, there is a high risk of unintentional overdose [9]. NSAID’s are drugs with analgesic and anti-inflammatory properties. They inhibit the enzyme cyclooxygenase (COX), which catalyzes the formation of prostaglandins and thromboxane. In the process of pain, prostaglandins cause local vasodilation and increased permeability of capillaries leading to edema. NSAID’s can have several potentially severe side effects, especially in older patients. Even a short course of NSAID’s can cause harm, such as cardiovascular events [10]. Annually, a considerable proportion (5.6%) of all unplanned admissions to Dutch hospitals is related to use of medication [11]. Internationally, these numbers are comparable. Half of these admissions are potentially preventable and the medications most often involved are, besides anticoagulants, NSAID’s. Reasons for hospitalization are mainly gastro-intestinal tract problems and cardiovascular problems. Of the potentially preventable admissions, 70% of the patients recover completely, however 6.3% die and 9.3% experience a disability after discharge. It is obvious that, besides the clinical need for the most effective treatment of pain in patients with an acute musculoskeletal trauma, the safety profile of both medications mentioned is equally important to make a judicious and evidence-based treatment plan.

The PanAM Study aims to deliver a high level of evidence (A2) answering the clinical question whether patients with acute musculoskeletal trauma should be treated with paracetamol, or NSAIDs, or both. The hypothesis is that paracetamol is not inferior to treatment with diclofenac, or the combination of both. Should paracetamol appear to be as effective as an NSAID in treating acute musculoskeletal injuries, use of NSAID’s could be reduced in this frequent disorder to minimize the number of NSAID-related adverse effects [12,13].

## Methods/design

### Ethics

The PanAM Study will be conducted in accordance with the principles of the Declaration of Helsinki, the Medical Research Involving Human Subjects Act (WMO) and ‘Good Clinical Practice’ (GCP) guidelines [14,15]. The protocol was approved by the IRB of the Academic Medical Center, and the Competent Authority.

### Study objectives

The primary objective is to determine which pharmacological pain management is best in adult patients with acute musculoskeletal trauma. The strategies that are compared are treatment with paracetamol + placebo diclofenac, diclofenac + placebo paracetamol and the combination of paracetamol and diclofenac. Secondary objectives are safety and adverse events, patient satisfaction and cost-effectiveness.

### Study design

The PanAM Study is a multi-center, double-blinded, randomized, non-inferiority trial.

### Setting

Patients will be included in two different settings. First: 24 hours per day and 7 days per week in the Emergency Department of a Dutch, urban, university hospital with 1032 beds in Amsterdam, The Netherlands. Annually, approximately 33000 patients are treated at this Emergency Department. Secondly, patients will be included in a General Practice in the vicinity of the hospital during office hours. Seven general practitioners work together in this General Practice with a patient population of approximately 10000 [16].

### Study population

All consecutive, adult patients with acute musculoskeletal trauma presenting to the Emergency Department or to the General Practice will be approached for inclusion. The allocation ratio in each of the comparison groups will be 1:1:1.

The inclusion criteria are:

adult patient, aged ≥18 years

non-penetrating limb injury, meaning a painful, acute strain, sprain or contusion of an extremity

trauma occurred within 48 hours before presentation

pain (mild, moderate and severe) scored with Numerical Rating Scale (NRS), regardless of amount of pain.

The exclusion criteria are:

previous treatment with analgesia for the same injury

self inflicted injury (“auto-mutilation”)

presence of wound, joint dislocation or more than one injury

presence of a fracture. An X-ray is done when estimated necessary by the treating pysician in order to exclude a fracture.

daily use of paracetamol and/or NSAID’s and/or other analgesia within two weeks before presentation

patients with chronic pain

previous adverse reaction or known allergy to paracetamol, NSAID’s or omeprazole

a known pregnancy

previous gastro-intestinal hemorrhage or perforation after NSAID use

active or recurrent peptic ulceration or peptic bleeding (2 or more evident episodes)

previous exacerbation of asthma after use of NSAID’s or acetylsalicylic acid

severe cardiac failure

liver cirrhosis

severe renal insufficiency (a known Glomerular Filtration Rate <30 mL/min)

bone marrow depression or blood dyscrasia (active or in past medical history)

combined use of angiotensin converting enzyme inhibitors (or angiotensin receptor blockers) AND diuretics [17]

physical, visual or cognitive impairment or non-Dutch language speaking (unable to use NRS, pain diary or EQ5D questionnaire)

All patients are treated in the Emergency Department or in General Practice and will not be admitted for surgery subsequently.

### The study consists of three different phases

#### Phase one

**Figure 1:**
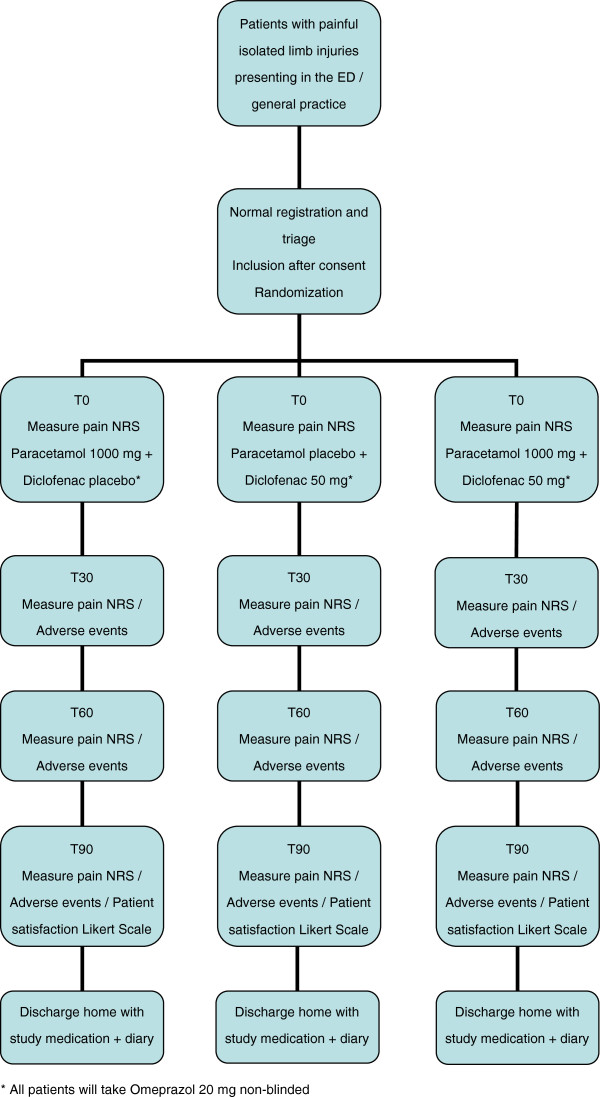
**Flow chart phase one of the study.** The flow chart depicts how patients are included in the study at the time of presentation in the Emergency Department or in general practice. T0 is the time of baseline measurement and administration of study drugs. T30 is 30 minutes after study drug administration, whereas T60 and T90 are 60 and 90 minutes respectively after study drug administration.

A patient presenting to the Emergency Department or the General Practice with an acute musculoskeletal trauma will be screened whether eligible for inclusion in the study. In the General Practice, this will be done by the physician and in the Emergency Department by a research assistant. Both have received a one-hour training of the research staff about all aspects of the study. If the patient is eligible, informed consent is obtained, randomization takes place and the patient is allocated to one of the three treatments. Figure 1 shows the study flow chart of phase one. Baseline characteristics are noted and pain in rest and with movement of the extremity is scored, using the NRS (T0). Patients verbally rate their pain intensity from 0 to 10; 0 being no pain and 10 being the worst possible pain. The patient receives two boxes of blinded study drugs. Box one contains 24 tablets paracetamol 500 mg or paracetamol-like placebo. Box two contains 9 tablets diclofenac 50 mg or diclofenac-like placebo. At T0, the patient takes two tablets from box one and one tablet from box two. All study drugs are taken orally. The combination of paracetamol-like placebo and diclofenac-like placebo is considered unethical (to fully withhold analgesia in patients with painful injuries). The possible treatment strategies with study drugs are:

1. paracetamol 1000 mg qid (*quater in die =* four times daily) + diclofenac-like placebo tds (*ter die sumendus =* three times daily)

2. paracetamol 1000 mg qid + diclofenac 50 mg tds

3. paracetamol-like placebo qid + diclofenac 50 mg tds

Besides these drugs, all patients take the proton pump inhibitor omeprazole 20 mg qd (*quaque die =* once daily) for prevention of gastric internal bleeding during three days. Pain is re-evaluated at 30, 60 and 90 minutes (T30, T60 and T90). At T90, patient satisfaction is scored using a 5-point Likert scale with two questions. The occurrence of adverse events is recorded and the patient is discharged home. Phase one ends here.

#### Phase two

**Figure 2:**
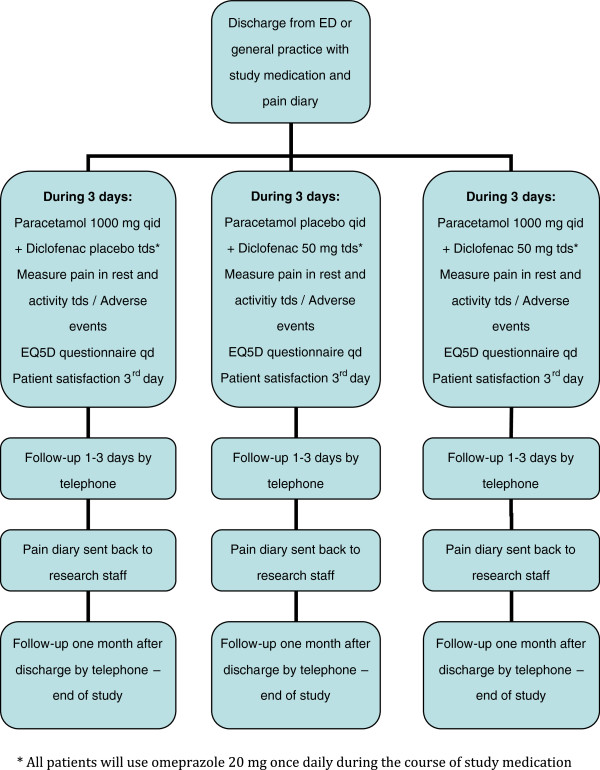
**Flow chart phase two and three of the study.** The flow chart depicts what study drugs patients take during three consecutive days, which parameters they record in the pain diary and when research staff contacts the patients. It shows the end of the study after the follow-up by phone after one month.

Figure 2 shows the study flow chart of phase two and phase three. After discharge, the patient takes home the study drugs, a pain diary and a stamped envelope. In the pain diary patients can score their pain each day three times a day during three days, in rest and with daily activities. During these three days the patient uses the same, blinded study drugs as mentioned in phase one. Patients can record adverse effects in the pain diary in predefined fields as well as in open fields. The EQ5D questionnaire is filled in daily and patient satisfaction using a 5-point Likert scale is recorded after three days [18]. In case additional analgesia is required, the patient can get a prescription of the treating physician. The physician will prescribe tramadol or another opioid, as it is unknown what blinded study drugs the patient is already using. Use of non-pharmacologic treatment (such as icepacks, bandages, crutches, etc) is documented. The patient sends the pain diary to the Emergency Department by post using the envelope.

#### Phase three

Phase three starts after the three days use of the study drugs and ends at 30 days after inclusion with a final follow-up by phone. The EQ5D health questionnaire is recorded, as well as the following parameters: use of additional pain medication, (late) occurrence of adverse events of study drugs, total days of leave from work and interventions needed. These interventions include physician visits, hospital admissions or surgery, care of a family caregiver, nursing at home, (paid) help needed in the household or temporary support in another way. The end of phase three is the end of the study for the participant.

### Endpoints

Primary outcome is between-group difference in decrease in pain (measured with NRS) at baseline and at 90 minutes after study drug administration in phase one of the study. Pain will be measured in rest and with movement of the extremity.

Secondary study parameters are

NRS at 30 and 60 minutes and pain measured during three consecutive days after discharge, in rest and with usual daily activity (walking, bathing, going to the toilet) three times daily.

proportional changes in pain; <33% or >33% decrease and Number Needed to Treat (NNT) to achieve 33% decrease in NRS.

occurrence of adverse events

patient satisfaction with pain relief in phase one and phase two, using a 5-point Likert scale with two questions.

need for additional pain medication in all phases and use of non-pharmacologic co-interventions.

analysis of quality of life and economic evaluation

baseline parameters: age, sex, medical history and medication use, date of injury and date of visit, mechanism of trauma, type and site of injury and use of non-pharmacological co-interventions.

### Randomization

After confirmation of eligibility for the trial and obtaining written informed consent, the patient is allocated to a blinded study drug treatment using a randomization list. The randomization list was created in advance by the Clinical Research Unit for statistical and epidemiological support in conjunction with the trial pharmacy. The trial pharmacy packed the study drugs in identical, blinded packages and labeled the packages with the randomization numbers. A fixed block size of 9 is used. Patients are stratified in subgroups younger and older than 60 years. After including a patient in the study, an online randomization module is used to obtain the randomization number (ALEA Software for Randomization in Clinical Trials, version 2.2, Copyright 2004 NKIAVL Amsterdam, NL). The study drug package labeled with this randomization number is picked up by the research assistant and the study starts for the participant. Patients, care providers and assessors of outcomes are all blinded for the assigned study drug.

### Study drugs

The study drugs are manufactured according to Good Manufacturing Guidelines (GMP) Annex 13. Management of study drugs is performed according to Dutch national law and Good Clinical Practice (GCP). Paracetamol and placebo paracetamol as well as diclofenac and placebo diclofenac have similar appearances.

### Sample size calculation and statistical analysis

The primary outcome (difference in NRS between groups) is expected to be distributed normally. In a pilot study we found a standard deviation of decrease in NRS score of 2.06. The equivalence limit is chosen at 0.75 to be certain that the value is well below the clinical relevant difference between NRS measurements of 1.3. This 1.3 difference in NRS is chosen, based on prior work by several investigators that identified the minimally clinically significant difference in pain as 1.3 units on an NRS [19,20]. As we want to assess non-inferiority two times (once between the paracetamol and diclofenac group and once between the paracetamol and combination group), we use a Bonferroni adjustment of the significance level to protect against type I error. Using this data, a one sided t-test with a significance level of 0.0125, an equivalence limit of 0.75 and an expected difference of 0 between two groups, will have 85% power to reject the null hypothesis that ‘paracetamol only’ is inferior to one of the other groups when the sample size in each group is 164. With three groups and taking into account an anticipated loss to follow-up of 10% we will need 547 patients totally. It is expected that this number of study subjects is reached within a year. In case the null-hypothesis, that paracetamol is inferior to the other two treatment strategies, will be accepted, a secondary analysis will be done. This secondary analysis will investigate superiority of diclofenac or the combination of both versus paracetamol. It can be undertaken with a one-sided T-test, a significance level of 0.0125 (Bonferroni adjustment) and 90% power to detect a clinical significant between group difference of 1.3 in NRS [21]. In each group, 64 patients are needed for this analysis, meaning that enough patients will already be included. Continuous data conforming to a normal distribution will be analyzed using one-way analysis of variance and Kruskal-Wallis test is used for continuous data without a normal distribution. Unpaired ordinal data will be analyzed using Chi Square test or the Fisher exact test. Paired ordinal data will be analyzed using generalized estimating equations (GEE models). A planned subgroup analysis will be done in patients older than 60 years, as these patients are more likely to develop adverse effects when using NSAID’s. Data will be analyzed using SPSS version 19.0 SPSS Inc., Chicago, IL.

### Economic evaluation

Economic evaluation will include costs-effectiveness analysis (primary outcome costs per unit decrease in pain) and cost-utility analysis (costs per quality adjusted life year (QALY)) from a social perspective with a time horizon to 1 month after discharge by follow-up by phone to measure relevant effects and costs. For analysis of quality of life, health outcomes will be assessed using Euroqol – EQ5D questionnaire during the first three days and after one month by telephone. Previous research has determined the utility of each observed health score profile on the EQ-5D based on the time trade-off elicitation technique during interviews with adults from the Dutch general population [22]. QALYs will be calculated by the product sum of the utility of each health state and the times in between the actual observations of those health states and the previous ones.

Direct and indirect medical and non-medical costs will be included in the economic evaluation, as well as health-related costs for patients and family and costs of loss of productivity. Medical costs will include the costs of initial evaluation of the patient, as well as follow-up needed and interventions needed (with all diagnostic and therapeutic procedures performed) when adverse events from the study drugs occur. Unit costing will be based on available national costing guidelines for health care research. Direct and indirect non-medical costs of, respectively, out-of-pocket expenses and production loss during the first month after sustaining the injury will be estimated. Indirect costs of production loss will be calculated with the friction cost method, based on the Dutch situation. Costs will be estimated for the base year 2013; price-indexing will be applied for unit costs originating from different calendar years.

### Monitoring

An Internal Medicine Physician with a specialization in Clinical Pharmacology and Acute Internal Medicine, who is not related to the study, is appointed as a safety monitor. The safety monitor is blinded. Adverse events are presented to the safety monitor and findings will be reported in a regular format to the principal investigator and the coordinating investigators. Monitoring visits for GCP compliance will be scheduled once a year at each study site and the following are included in the monitoring plan: inclusion speed and drop out percentage; presence and completeness of the research files; informed consents; in- and exclusion criteria; Source Data Verification; occurrence of Serious Adverse Events (SAE’s) and verification of the procedures following this; patient instructions and instructions for execution of study procedures and management of study medication according to GCP and Dutch national law.

As the study is estimated to be a study of low risk (approved drugs in regular doses used for a short period in a population that uses the drugs every day in current clinical practice), there is no need to install a formal Data Safety Monitoring Board (DSMB).

## Discussion

Acute musculoskeletal trauma occurs frequently in The Netherlands as well as in other Western Countries [1,2]. There is no conclusive evidence about pharmacologic treatment of these injuries and whether paracetamol is as effective as an NSAID. A systematic review from 2010 analyzed two Randomized Controlled Trials (RCT’s) comparing paracetamol to NSAID’s in patients with musculoskeletal disorders [3]. The first was a trial from Hong Kong comparing three days of oral paracetamol 4000 mg, indometacin 75 mg, diclofenac 75 mg and paracetamol 4000 mg + diclofenac 75 mg in 300 patients with musculoskeletal injuries of back, neck and extremities showing no significant difference in pain relief in the acute phase and three consecutive days [23]. It is questionable whether these results can be extrapolated; diclofenac was dosed lower than in daily practice in The Netherlands and other Western countries. The authors concluded that other studies would be required to address this issue. In the second RCT 260 patients with ankle sprains were treated with paracetamol 3900 mg or ibuprofen 1200 mg daily [24]. There was no significant difference in pain reduction. Two other RCT’s from 2007 and 2011 found no significant difference in decrease in Visual Analogue Scale (VAS) in 100 and 90 patients, treated for ankle sprains five and ten days respectively with diclofenac 150 mg or paracetamol 1500 mg daily [25,26]. It is questionable whether these results can be extrapolated, as the daily dose paracetamol used in The Netherlands is much higher. Direct comparison studies between paracetamol and NSAID’s in other clinical problems than musculoskeletal trauma show varying results [27]. NSAID’s seem more effective in dental and menstrual pain, but both drugs provide equivalent analgesia in orthopedic surgery and tension headache.

The current study aims to provide evidence whether paracetamol is or is not inferior to diclofenac or a combination of paracetamol and diclofenac in adult patients with acute musculoskeletal trauma. Diclofenac 50 mg tds was chosen, because this drug in this dose is common practice in the Netherlands and frequently used in patients with acute musculoskeletal trauma. The combination of paracetamol and diclofenac is also often prescribed. In literature, this drug combination is frequently used in treating patients with postoperative pain [28,29]. The reason for combining two analgesic drugs with different pharmacological modes of action is that they might work supra-additively; their analgesic effects might enhance each other. In daily clinical practice, the superiority of the combination of paracetamol and NSAID’s has not been proven so far [30]. The authors of a systematic review from 2010 concluded that the combination of paracetamol and diclofenac might give better results with respect to pain relief [31]. However, this review included studies with differences in methodology that can significantly influence the results in pain score. At first, the review included studies in patients with pain in the peri-operative setting, where analgesic drugs are frequently administered before patients experience pain. Secondly, in some of the studies included in this review drugs were administered rectally instead of orally and the absorption and the rate of absorption differ between rectal and oral use. Finally, in several studies included, paracetamol and diclofenac were used in different dosages. The results of this systematic review therefore cannot be used in the daily clinical, non-operative setting, from which the patients in the PanAM Study are selected.

The non-inferiority design, with the null hypothesis that there is a significant negative difference in NRS, is chosen as study design, as this is thought to harvest study results that will be the most relevant to daily clinical practice. When paracetamol turns out to be not inferior to diclofenac or the combination of paracetamol and diclofenac (i.e. the alternative hypothesis is accepted), sufficient evidence is supplied that NSAID’s should not be prescribed to patients with acute musculoskeletal trauma. This will hopefully lead to a decrease in NSAID-related adverse effects. When the study results will be such that the null hypothesis will be accepted, a secondary analysis will take place to investigate whether diclofenac or the combination of both drugs is statistically superior to the use of paracetamol.

In the PanAM Study, patients are included in an Emergency Department and in a General Practice. By selecting patients in these different centers, the results of the PanAM Study will be easily generalized to daily clinical practice, as patients with acute musculoskeletal trauma present to these different centers daily.

The occurrence of adverse drug effects could have been chosen as primary endpoint of the study, as it would be expected that NSAID’s would have more adverse effects than paracetamol, used in therapeutic dosages. Instead, between- group difference in decrease in NRS is chosen as primary outcome. It is unknown which of three studied treatment strategies is the most effective in patients with acute musculoskeletal trauma. Having used the occurrence of adverse effects as primary endpoint would have resulted in a very large population needed for the study. Proportional changes in pain are used as secondary endpoints. A decrease in NRS of more or less then 33% and the NNT to achieve 33% decrease in NRS are used. In 2009, Chang mentioned that there is no consensus about the best way to measure efficacy of different pain regimens [32]. By adding these secondary endpoints, we would like to determine whether the findings of the primary outcome will be concordant with these secondary outcomes.

In the PanAM Study, all subjects receive omeprazole for gastric protection. The bias of safety results that will inevitably occur is not relevant to our results, as we primarily aim to measure effectiveness. We realize that the adverse effects of diclofenac measured in this trial are underestimated. This strategy is the only ethical way to include elderly patients in this study, as the Dutch national guideline “Use of NSAIDs and prevention of peptic injury” advises prescribing proton pump inhibitors when NSAID’s are used in all patients older than 60 years [33]. The guideline also states that a proton pump inhibitor should be administered when prescribing NSAID’s in patients with a past history of peptic ulcer or untreated H. pylori infection; use of anticoagulants; severe rheumatoid arthritis; heart failure or diabetes; use of corticosteroids or Selective Serotonine Re-Uptake Inhibitors (SSRI’s). As we aim to treat all participants exactly the same way to prevent co‒intervention bias, we choose to administer omeprazole to all patients in this study. The alternative would be to exclude all patients with higher risk of NSAID-related GI-events, however, as we are highly interested in the group of elderly patients in a subgroup analysis, this is not feasible.

The PanAM trial will focus on the question which pharmacologic pain treatment is best in patients with acute musculoskeletal trauma and will highlight the subgroup of patients older then 60 years. This group seems vulnerable when using NSAID’s as the occurrence of adverse events seems to increase with increasing age.

## Conclusion

The PanAM Study is a multi-center, double blind, non-inferiority trial that aims to answer the question whether paracetamol is not inferior to diclofenac or paracetamol + diclofenac in the treatment of pain sustained from acute musculoskeletal trauma.

### Prospective

Inclusion at the Emergency Department started July 2013. Inclusion at the General Practice is planned to start October 2013. The study is expected to last for one year.

## Abbreviations

CA: Competent authority; CCMO: Central Committee on Research Involving Human Subjects (in Dutch: Centrale Commissie Mensgebonden Onderzoek); COX: CycloOxygenase; DSMB: Data Safety Monitoring Board; EudraCT: European Union Drug Regulating Authorities Clinical Trials; GCP: Good clinical practice; GMP: Good manufacturing practice; NRS: Numerical rating scale; NSAID’s: Non-steroidal anti-inflammatory drugs; QALY: Quality adjusted life year; qd: *quaque die* = once daily; qid: *quater in die* = four times daily; RCT: Randomized controlled trial; SAE: Serious adverse event; tds: *ter die sumendus* = three times daily; VAS: Visual analog scale; WMO: Medical Research Involving Human Subjects Act (in Dutch: Wet Medisch-Wetenschappelijk Onderzoek met Mensen).

## Competing interests

ML Ridderikhof, MD, is a PhD Student at the departments of Anesthesiology and Traumatology, employed by the Academic Medical Center as a staff member of the department of Emergency Medicine. As a researcher, he is supported by a grant from ZonMW, the Netherlands organization for health research and development with grant number: 836011015. All authors declare that they do not have any competing interests.

## Authors' contributions

MR designed and coordinates the study, created the paper as well as the electronic case report forms and drafted the manuscript. MR was funded partially from the ZonMW grant. PL, AH, WG, JL, MK, MD, MH and CG helped designing and coordinating the study. AH helps coordinating the study in the General Practice. WG and MD helped with the statistical and economic analysis. MK is responsible for coordination and distribution of study medication in both study sites. All authors revised and corrected earlier versions of the manuscript critically. All authors read and approved the final version of this manuscript.

## Pre-publication history

The pre-publication history for this paper can be accessed here:

http://www.biomedcentral.com/1471-227X/13/19/prepub
